# Cathodic hydrogen production by simultaneous oxidation of methyl red and 2,4-dichlorophenoxyacetate aqueous solutions using Pb/PbO_2_, Ti/Sb-doped SnO_2_ and Si/BDD anodes. Part 1: electrochemical oxidation

**DOI:** 10.1039/d0ra03955a

**Published:** 2020-10-21

**Authors:** José Eudes L. Santos, Djalma R. da Silva, Carlos A. Martínez-Huitle, Elisama Vieira dos Santos, Marco A. Quiroz

**Affiliations:** Universidade Federal do Rio Grande do Norte, Instituto de Química Campus Universitário 3000 CEP 59078970 Natal RN Brazil carlosmh@quimica.ufrn.br marco.quiroz@ccet.ufrn.br

## Abstract

In this work, the electrochemical oxidation of the Methyl Red (MR) dye and the herbicide sodium 2,4-dichlorophenoxyacetate (2,4-DNa) was investigated on Si/BDD, Pb/PbO_2_ and Ti/Sb-doped SnO_2_ anodes in aqueous acidic medium by applying 30 mA cm^−2^ at 298 K. The electrochemical experiments were carried out in a two-compartment electrochemical cell separated through a Nafion® membrane (417 type) in order to use two types of supporting electrolyte to measure the elimination of the organic compound, the hydrogen production and the amount of oxygen produced during the oxidation of the pollutants. Although the main goal of this study is to understand the relationship between both processes, the evaluation of the current efficiencies (*η*) is a key parameter to determine the anodic oxidative capacity to degrade the proposed pollutants. The results clearly showed that MR and 2,4-DNa can be oxidized on Si/BDD, Pb/PbO_2_ and Ti/Sb-doped SnO_2_ anodes; however, significant variations in the oxidation level and *η* are achieved. Thus, although the MR solutions were completely discolored in all cases, only on the Si/BDD anode was MR oxidized to carboxylic acids in less than 15 min of electrolysis time. On Pb/PbO_2_ and Ti/Sb-doped SnO_2_ electrodes, the discoloration was slower and the oxidation was quasi-completed, leaving in solution some organic by-products, such as 2-aminobenzoic acid and/or *N*,*N*′-dimethyl-*p*-phenylenediamine, in the fixed electrolysis time. The behavior observed during the elimination of 2,4-DNa is due to its difficulty in degrading the chlorine groups in its aromatic ring which makes 2,4-DNa a more stable molecule. In the first oxidation stage, 2,4-dichlorophenol (2,4-DP) is produced in all cases, but on Si/BDD, this intermediate is quickly consumed. From the polarization curves and Tafel analysis, a reaction scheme for the formation and consumption of 2,4-DP was proposed.

## Abstract 2

In this work, the electrochemical oxidation of a dye and an herbicide was investigated on non-active anodes in aqueous acidic medium in order to understand its effect to produce simultaneously hydrogen as eco-friendly energy alternative.

## Graphical abstract

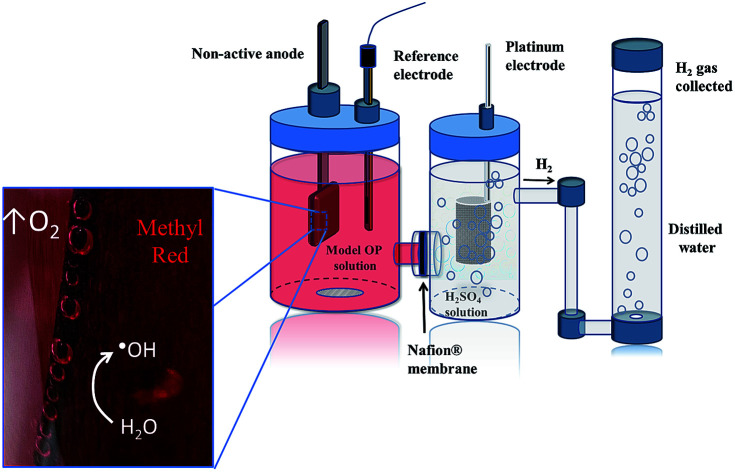

## Introduction

In the wake of the problems of climate change, increased global energy demand, and variations in oil production and its prices, the search for alternative sources of energy has become a priority research issue. Since the mid-twentieth century, several options have been studied, and some of them have been developed on the scale of industrial application, but not enough to cover the demand or even partial replacement of fossil fuels. At this point, it is important to recognize that the solution will not come from the development of single devices that cover all energy generation needs, that is sustainable and, of course, compatible with the environment. Rather, the current trend is to design hybrid devices that combine various energy generation techniques, where each part of the device develops an efficient specific activity. In this sense, using primary sources of energy,^1,2^ such as wind energy^3^ and/or photovoltaics,^4^ to operate hydrogen gas generators, an almost inexhaustible source of clean fuel could be available to support the future development of industry and maintain the form that society develops.^5^

Highlighting hydrogen, as an energy carrier, is important, not only because it is a clean and abundant fuel, as a chemical component of the water; but due to its low equivalent weight it has the highest energy density per unit mass (33.33 kW h kg^−1^), which is triples in value to the observed for natural gas (82–93% CH_4_; 10.6–13.1 kW h kg^−1^), or any other fossil fuel. Unfortunately, the current production of hydrogen gas continues to be *via* thermocatalytic conversion methods.^5,6^ Nevertheless, since the prediction by Fujishima and Honda work in 1972^7^ about the water splitting by electrolysis as well as by photoelectrolysis; these approaches are considered the most relevant in the hydrogen economy. However, the energy required to dissociate the water molecule is still an unresolved problem. For this reason, this energy can be obtained by a source associated to the electrocatalytic system or by a tool that can supplement the system internally; and consequently, minimizing the costs to make profitable the technology. In this regard, it was recently proposed the electrochemical oxidation process as an alternative to produce hydrogen gas by using methanol,^8^ as a sacrificial compound, with a proton exchange membrane electrolysis cell (PEMEC). Methanol oxidation on a Pt–Ru(1 : 1)/C anode allowed to diminish significantly the energetic requirements for water electrolysis, achieving 14.5 cm^3^ of hydrogen production after 20 min of electrolysis of a 2 M methanol solution by applying 100 mA. This study also allowed to establish that, the hydrogen production was only dependent on the applied current, but it was affected by the deactivation of the anodic electrocatalyst due to the CO formation from methanol oxidation. Therefore, the use of a sacrificial analyte to reduce the amount of energy expended in the water electrolysis seems to be a good alternative.

Another option is to consider the hydrogen production as a subsequent stage of a primary stage of greater interest, such as the case of the electrooxidation of organic pollutants.^9^

Recently, several works have shown that the wastewater treatment by using electrochemical technologies could have a dual-activity alternative *via* the elimination of organic pollutants (R) from wastewaters and simultaneously, producing hydrogen gas in economical-acceptable efficiencies.^10^ According the existing literature,^11–13^ R degradation in aqueous solutions is achieved at higher efficiencies when non-active anodes are used because the oxidative process take places with the participation of hydroxyl radicals (˙OH) from water electrolysis, and/or persulfate (S_2_O_8_^2−^) and ion radical sulfate (SO_4_^−^˙) when boron doped diamond (BDD) is used as anode in a sulfate (SO_4_^2−^) medium.^14^ The oxidation level of R depends on the anodic material (M), the applied current density (*j*_app_) and the chemical composition of the treated solution.^11^ Water discharge on the anode to produce ˙OH is the initial step in the electrochemical oxidation process and these species can are adsorbed (chemically or physically) on the anodic surface:

1 M + H_2_O_(l)_ → M(˙OH) + H_(ac)_^+^ + e_aux_^−^

where e_(aux)_ represents the electrons transferred to the auxiliary electrode. For those anodic materials where the ˙OH are weakly adsorbed (physically adsorbed), such as PbO_2_, SnO_2_ and BDD (commonly classified as non-active anodes),^15^ electrochemical combustion or electrochemical mineralization of R is attained, if the electrolysis time is enough:

2 M(˙OH) + C_*m*_H_*n*(ac)_ → M + *m*CO_2(g)_ + *n*H_2_O_(l)_ + *n*H_(ac)_^+^ + *y*e_aux_^−^

Meanwhile, for anodic materials (M) where the ˙OH are chemically adsorbed on their surface, an oxide-structure (MO_*x*+1_) is formed, then, the oxidation pathway leads to the electrochemical conversion of the R.

3 MO_*x*+1_ + R_(ac)_ → RO + MO_*x*_

In both cases, the oxidation of R is attained in parallel with the evolution of molecular oxygen (O_2_), according to the reactions:4
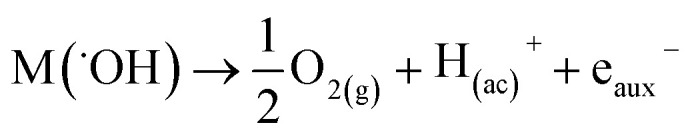
5
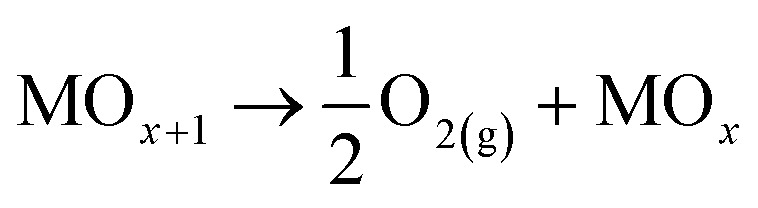


At this point, it is important to remark that, the electrons produced in the reactions (1), (2) and (4), electrochemical stages at non-active electrodes, flow through the cell to the auxiliary (aux) electrode (Pt if possible), where they are involved in the complementary reaction of hydrogen evolution,6
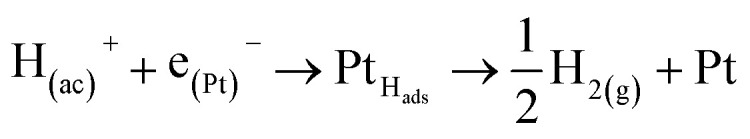


If the process is carried out in undivided electrochemical cells, either batch or flow type, both the hydrogen gas and the oxygen gas produced at the cathode and anode materials, respectively, are released from the cell as a mixture.^11^ Additionally, the gas mixture will also contain CO_2_ gas due to the electrochemical mineralization of R. Then, if the target is producing and collecting hydrogen gas, associated with the oxidation of pollutants; the use of double-compartment cells (or divided electrochemical cells) should be the best strategy because at the cathodic compartment will be produced hydrogen gas while at the anodic compartment will be oxidized the organic pollutants but oxygen will be also produced.

Therefore, the aim of this work is to demonstrate that the electrochemical oxidation of two common organic pollutants (methyl red dye and 2,4-DNa herbicide in aqueous solution) using a divided cell with non-active anodes (Pb/PbO_2_, Ti/SnO_2_ and Si/BDD) is an eco-sustainable and efficient alternative to produce hydrogen gas in the cathodic compartment (Pt electrode in 0.25 M H_2_SO_4_ solution). Firstly, the electrochemical oxidation process was investigated and discussed in order to prove that the degradation of both model organic compounds is efficiently achieved. Production of hydroxyl radicals and by-products was kinetically explored as well as the mineralization and current efficiencies were also estimated. Non-active anodes were chosen because these electrocatalytic materials promote a complete mineralization of organic matter in anodic compartment,^12,16^ and because no attempts have been published in the literature to produce hydrogen gas coupled with electrochemical oxidation by using these anodic materials in divided cell.

## Experimental section

### Chemicals

Ultrapure water (18 MΩ) was obtained through a Simplicity water purification system. Chemicals were of the highest quality commercially available and were used without further purification. Methyl Red (MR), 2,4-dichlorophenoxyacetic sodic salt (2,4-DNa) (puriss. p.a. ≥99.5%), *N*,*N*-dimethyl-*p*-nitrosoaniline (RNO) (purum p.a. ≥98.0%), 2,4-dichlorophenol (2,4-DP), glycolic acid (GAc), Na_2_SO_4_ and H_2_SO_4_ (puriss. p.a. 95–97%) were purchased from Fluka and/or Aldrich companies. The model organic compound solutions were prepared dissolving an amount (in mg) of organic compound (in the range of 10–100 ppm) in ultrapure water containing 0.5 M H_2_SO_4_ or 0.5 M Na_2_SO_4_ as supporting electrolyte.

### Electrode materials

Pb/PbO_2_ electrode was prepared growing the anodic oxide at a current density of 50 mA cm^−2^ during an electrolysis time of 90 min in a 10% (v/v) H_2_SO_4_ solution at 25 °C in order to oxidize the lead surface into PbO_2_. More details concerning anode preparation and characterization are given elsewhere.^17–19^ SnO_2_-coated titanium (Ti/SnO_2_) electrode^20,21^ was prepared by a sol–gel technique, which consisted of the following steps: preparation of precursor solution, its brushing onto a pre-treated titanium base and drying at 50 °C. The whole process was repeated 10 times. BDD films were provided by CSEM (Neuchâtel, Switzerland) and synthetized on a conductive p-Si substrate (1 mm, Siltronix) *via* a hot filament using the chemical vapor deposition technique (HF-CVD).^22^ This procedure gave a columnar, randomly textured, polycrystalline diamond film, with a thickness of about 1 μm and a resistivity of 15 mΩ cm (±30%) onto the conductive p-Si substrate.

### Electrochemical measurements

The electrochemical experiments were developed in a two-compartment cell, both of them separated by a Nafion® membrane of 350 and/or 417 type with the opaque face towards catholyte solution. Hydrogen gas was measured by using an inverted burette directly connected to the cathodic compartment which has a capacity of 100 mL; whereas the anodic compartment having a capacity of 200 mL. A Pt-mesh of 37 cm^2^ of real surface area was used as cathode, in all experiments. The real surface area of Pt was calculated by the well-known method of hydrogen adsorption.^23–25^ Cyclic voltammetry was developed in a single three electrode cell using 0.5 M H_2_SO_4_ solution as supporting electrolyte, a Hg/Hg_2_SO_4_/K_2_SO_4_(sat) (MSE) and Pt wire as reference and auxiliary electrodes, respectively. Pb/PbO_2_, Ti/SnO_2_ and Si/BDD were used as the anodes and a MSE or an Ag/AgCl (3 M) was used as the reference electrode. The anolyte consisted of an well-known amount of organic compound (in the range of 10–100 ppm) in 0.5 M H_2_SO_4_ or 0.5 M Na_2_SO_4_ solution as supporting electrolyte, whilst 0.25 M H_2_SO_4_ was chosen as the catholyte in all experiments. The concentration of the organic compound was not greater than 100 ppm in order to cover the level of concentration allowed by international regulations.^26^ The temperature of the electrolyte was fixed at 25 °C and maintained constant by using a water thermostat; also stirring rate was kept almost constant (350 ± 50 rpm) by using a magnetic stirrer. Under these experimental conditions, the estimated mass transfer coefficient in the cell, determined using the ferric/ferrocyanide couple, was 2 × 10^−5^ m s^−1^. The current density for the electrolysis (*j*_app_) was kept at 30 mA cm^−2^ with an Autolab model PGSTAT30 potentiostat–galvanostat. The electrolysis time was established at 60 min for comparison purposes, although in some cases it was extended until 120 min. After each run, both the cell and the anode were washed thoroughly with doubly (2–5 MΩ cm) and threefold distilled water (16–18 MΩ cm). During electrolysis, 3 mL samples were taken to follow the organic compound decay through UV-vis spectrophotometry at 520 nm, 440 nm and 285 nm wavelengths for MR, RNO and 2,4-DNa detection, respectively, with a Analytik Jena spectrophotometer.

### HPLC analysis

A Varian Model 9050/9012 HPLC equipped with a Waters Spherisorb 5 μm ODS2 (150 mm × 4.6 mm) reversed-phase column and a variable wavelength UV-vis detector was used to determine 2,4-DNa and by-products concentrations during various stages of the electrolysis. The most suitable mobile phase was 50/50 acetonitrile/H_2_O (2% CH_3_COOH) at a flow rate of 0.8 mL min^−1^, a column temperature of 30 °C and at an analytical wavelength of 288 nm. In all cases a 20 μL sample of electrolyzed solution was injected.

## Results and discussion

### Electrochemical characterization of the Pt electrode used as cathode

**Fig. 1:**
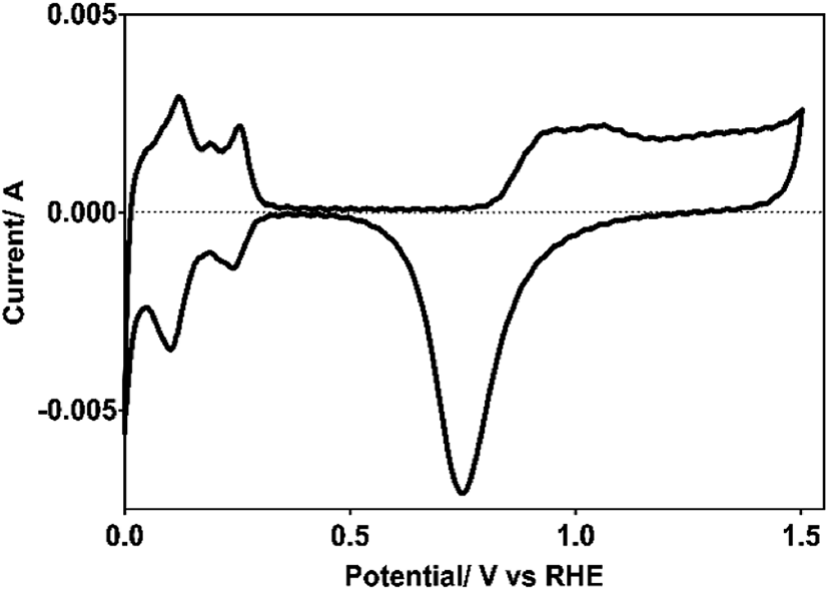
Voltammogram of the Pt electrode used as cathode, recorded in 0.5 M H_2_SO_4_ as supporting electrolyte at *υ* = 50 mV s^−1^ and at 298 K.

Pt electrode used as cathode for hydrogen production was electrochemically characterized by cyclic voltammetry (CV), both to verify the cleanliness of the exposed surface, and to determine its real surface area.^24,25^ Cleanliness was tested by the absence of Faraday currents in the double layer region (∼0.4–0.8 V/NHE) and the active surface area by the expended charge to desorb the hydrogen previously adsorbed (H_ads_) in the 0.4–0.088 potential range (*vs.* NHE), as shown in Fig. 1.

From the desorption hydrogen charge (*Q*_H,d_) measured between 0.08 and 0.47 V/NHE, using a standard charge for a polycrystalline Pt surface of 210 μC cm^−2^ and a coverage factor by hydrogen at 0.08 V/NHE of 0.77, the real surface area of the Pt electrode used here was of 37 cm^2^.

### Bleaching of *N*,*N*-dimethyl-*p*-nitrosoaniline (RNO) solutions as indicator of the anodic ˙OH production

Main advantage of an advanced oxidation process is the ability of the anode to act as a very active source to produce the primary oxidant species, free-hydroxyl radicals (˙OH), which are electrogenerated from water discharge (7):

7 H_2_O → ˙OH + H^+^ + e^−^

This strong oxidizing agent (*E*_o_ = 2.8 V/SHE), attack almost any organic molecule, is non-selective and it has a high electron affinity. Therefore, the degradation products can be partially oxidized to intermediates or to mineralization products (*i.e.* carbon dioxide and water).

**Fig. 2:**
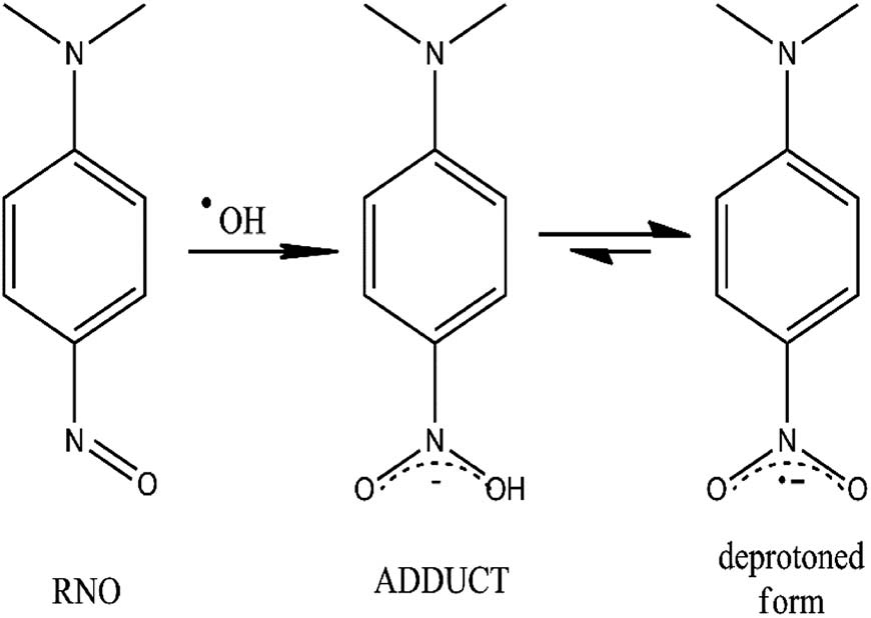
Chemical scheme for the scavenger process of ˙OH radicals by the RNO molecule and their equilibrium step between the adduct and the deprotoned form.

In this study, the production of ˙OH was tested in Pb/PbO_2_, Ti/SnO_2_ and Si/BDD electrodes by the bleaching of *N*,*N*-dimethyl-*p*-nitrosoaniline (RNO) solutions, reaction^27^ shown in Fig. 2.

RNO can be spectrophotometrically monitored at 350 nm or at 440 nm which are the characteristic wavelengths of RNO in acid and alkaline medium, respectively. Moreover, RNO is not electroactive with respect to anodic oxidation and it exhibits high reaction rate with ˙OH.^28^ By using, a kinetic model of pseudo-first order for the RNO discoloration process on the studied anodes,8
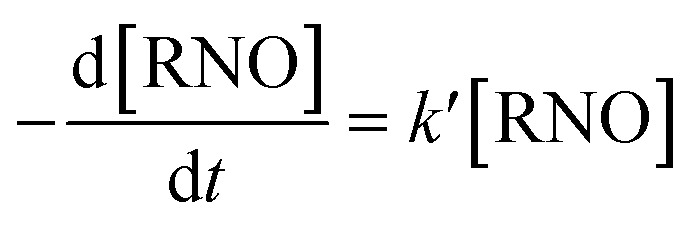
where *k*′ = *k*[˙OH] represents the pseudo first-order rate constant, the calculated *k*′ values respect to the time for each one of the electrodes studied as anodes, were obtained, Table 1.

**Table 1:** Pseudo-first order rate constants for the RNO discoloration process in 0.05 M Na_2_SO_4_ + 1.85 × 10^−5^ M RNO as calculated from integrated form of eqn (9) for each one of the electrodes studied as anodes

Anode	*k*′ in min^−1^	
	pH = 3.1	pH = 9.2
Pb/PbO_2_	0.131	0.0296
Ti/SnO_2_	0.0102	0.0125
Si/BDD	0.268	0.0483

These results confirm that the electrocatalytic activity to produce ˙OH, by the non-active anodes studied here, is quite satisfactory and that the bleaching RNO reaction can be assumed as a pseudo-first order reaction as a function of [RNO]. This fact means that the bleaching RNO reaction is either independent of the ˙OH concentration or that this ˙OH concentration is in a large excess and remains constant during the bleaching RNO reaction. According to the reaction in Fig. 2, the first assumption in not feasible, therefore, the rate constant values must be associated with a greater or lesser availability of ˙OH in solution. As previously discussed, at non-active anodes, the concentration of ˙OH radical at the interfacial region depends on the strength at which they are physically adsorbed after the electronic transfer of their formation. But, while the electrolytic process is maintained, the production of ˙OH will be continuous, maintaining more or less constant interfacial ˙OH concentration, despite the consumption made to discolor the RNO. Thus, the assumption of a pseudo-first order kinetic for the RNO discoloration reaction is justified.

### UV-vis spectroscopic characteristics of 2,4-DNa and MR

Since oxidation studies of organic pollutants are regularly followed by spectroscopic techniques,^29,30^ it is a recommended practice to establish previously their spectroscopic characteristics in order to properly interpret the results of their electrochemical oxidation.

**Fig. 3:**
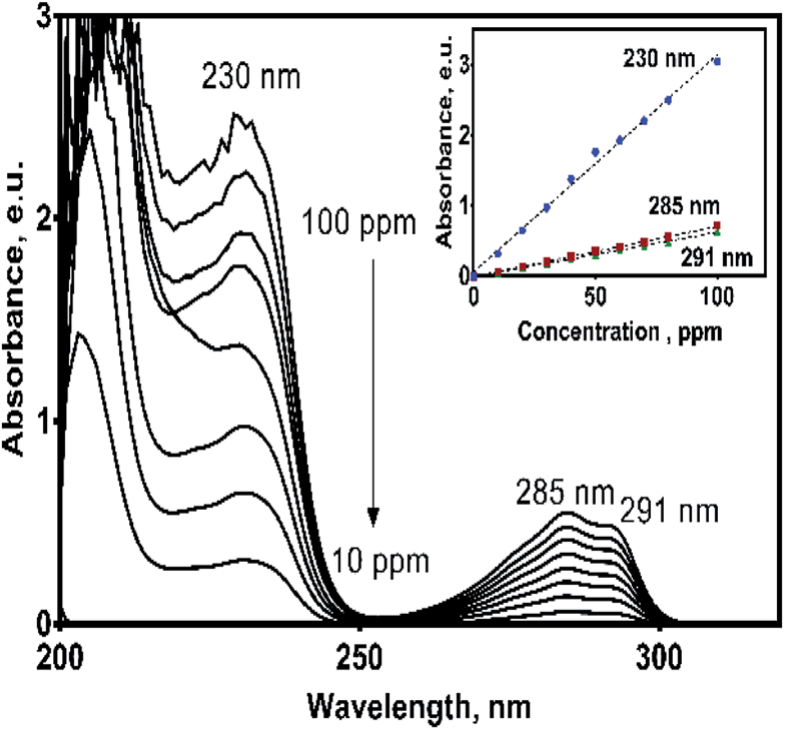
UV absorption spectra of 2,4-DNa in 0.5 M Na_2_SO_4_ (pH 3) solution as a function of 2,4-DNa concentration in the range of 10–100 mg L^−1^. Inset: Beer law applied to each one of absorption bands considered.

UV spectrum of the 2,4-DNa exhibits essentially three absorption bands whose wavelength values show an important bathochromic shift towards higher values due not only to the strong substitution of the benzene ring but also to the influence of the aqueous medium used as solvent. In 0.5 M Na_2_SO_4_ + *x* mg L^−1^ 2,4-DNa solutions, the observed wavelength values were ∼210, 230 and 285-291 nm, being these values associated to the second-primary ′B band, the primary ′L_a_ band and the benzenoid absorption bands, respectively.^31,32^ UV band observed at 204 nm can be associated to the nπ* band of the acetate group into the 2,4-DNa molecule. Fig. 3 shows UV spectra of 2,4-DNa as a function of 2,4-DNa concentration as well as the corresponding Beer's law applied to each absorption band observed (inset in Fig. 3) for the phenyl group and for which the following molar absorptivity (*ε*) values were calculated: *ε*_291_ = 1533.4, *ε*_285_ = 1725.4 and *ε*_230_ = 7484.9 L mol^−1^ cm^−1^. From these results, the wavelength at 285 nm was selected as the one to which the quantitative analysis of 2,4-DNa must been performed, and although it is not the most intense, it is the most adequate to follow the evolution of the aromatic ring.

**Fig. 4:**
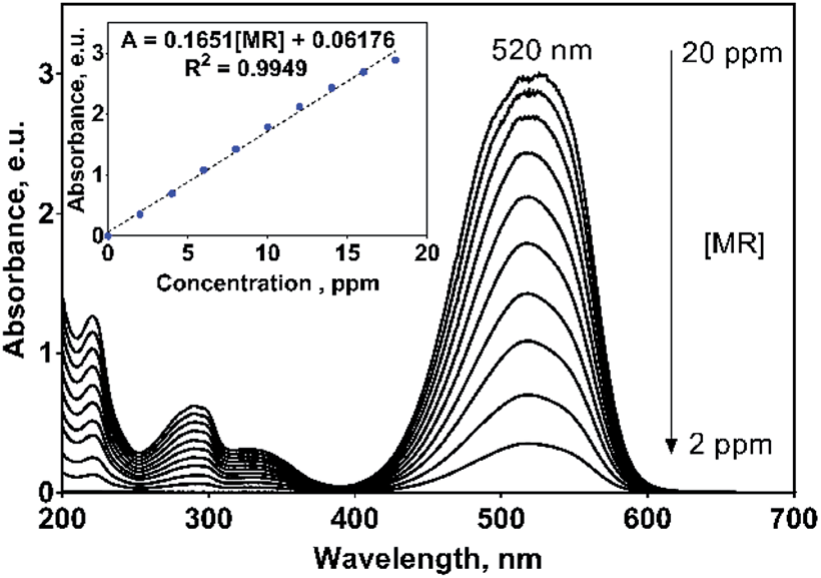
UV absorption spectra of methyl red (MR) in 0.25 M H_2_SO_4_ solution as a function of MR concentration in the range of 2–20 mg L^−1^. Inset: Beer law applied to the principal absorption bands located at 520 nm.

On the other hand, MR dye showed an UV-vis spectrum with their main absorption band at 520 nm (n → π* transition of –N=N– group) and three UV bands at 220, 290 and 335 nm (π → π* transition) related to the aryl groups of the MR molecule, Fig. 4.^33^ The absorption band at 520 nm is the responsible to the red color of this azo dye solution. For monitoring purposes, the band at 520 nm was chosen and for which a great molar absorptivity (*ε*) value of 44 461.4 L mol^−1^ cm^−1^ was estimated (inset in Fig. 4). It is important to remark that in both cases, the UV-vis profiles uniformly varies with the 2,4-DNa and MR concentrations, which is not discussible since the molecular structure does not change with dissolution, but it is a point to take into consideration in the oxidation processes where the molecular structure could be significantly modified or even disappear.

### Electrocatalytic discoloration of MR

**Fig. 5:**
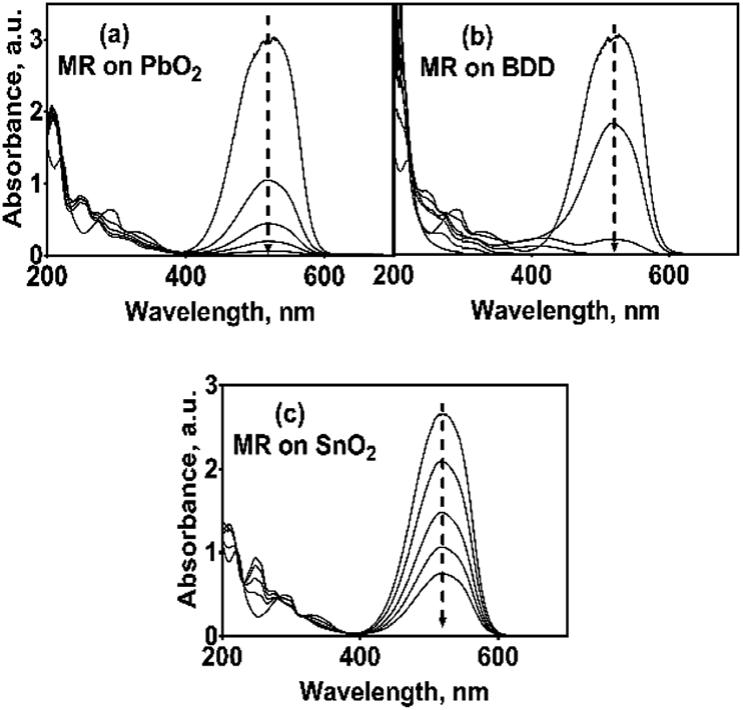
UV-vis spectra for 20 mg L^−1^ MR solutions in 0.5 M H_2_SO_4_ electrolyzed at 30 mA cm^−2^ and 298 K, as a function of the electrolysis time and the material of anode: (a) Pb/PbO_2_, (b) Si/BDD and (c) Ti/Sb-doped SnO_2_.

The discoloration of MR during the electrolysis of 0.25 M H_2_SO_4_ + 20 ppm MR solutions by applying 30 mA cm^−2^ for the different studied anodes, is showed in Fig. 5a–c, through the corresponding UV-vis spectra.

UV-vis spectra showed an important decrease of the absorption band at 520 nm and, therefore, a rapid discoloration of the solution with the electrolysis time was registered. Furthermore, after 120 min, both Si/BDD and Pb/PbO_2_ anodes not only discolor the MR solutions but also degrade the MR to carboxylic acids,^34,35^ as suggested by the abatement of the spectral UV region between 260–350 nm, and the presence of the UV band at about 204 nm. For Sb-doped SnO_2_ anode, the discoloration is slower and a small UV band at 250 nm appears, which is probably related to the formation of the 2-aminobenzoic acid and/or *N*,*N*′-dimethyl-*p*-phenylenediamine as by-products of the first oxidation stage, eqn (9). However, this UV band tends to disappear with the electrolysis time.9
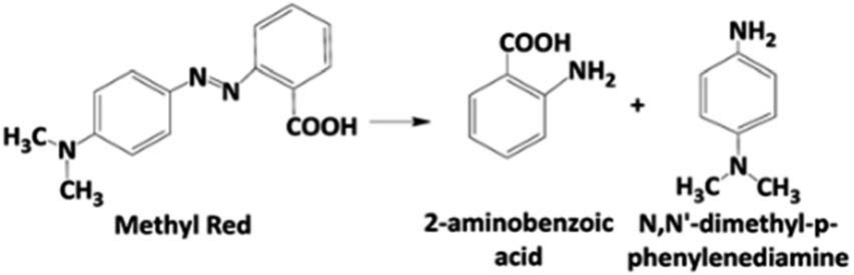


Certainly, these two intermediates have already been reported as products of the first stage of biodegradation of azo dyes, between them MR.^36^ Both aromatic amines are colorless compounds which follow a subsequent degradation by hydroxylation and ring opening to form between others the aliphatic primary and secondary amines,^37^ as confirmed by HPLC analysis.

**Fig. 6:**
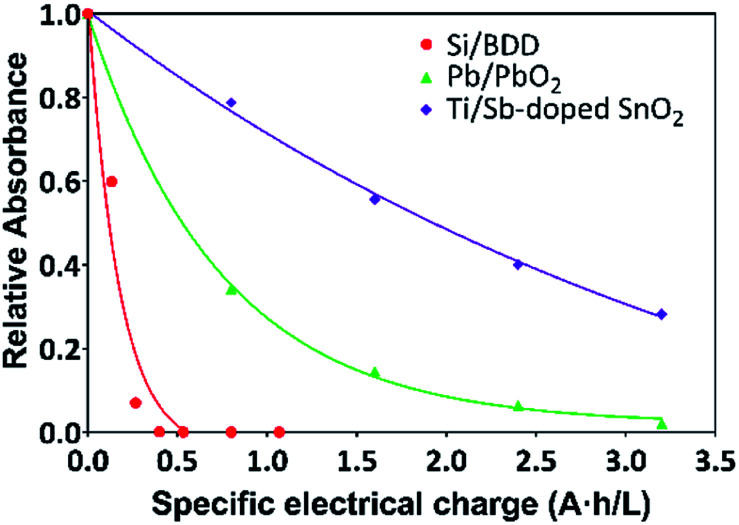
Plots of the relative absorbance of MR again specific electrical charge (A h L^−1^) for solutions containing 20 mg L^−1^ MR in 0.5 M H_2_SO_4_ at 30 mA cm^−2^ and 298 K: 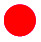 Si/BDD, 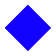 Ti/Sb-doped SnO_2_, 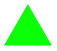 Pb/PbO_2_.

Thus, the variation of the relative absorbance of the MR solutions as a function of the specific electrical charge *Q* (A h L^−1^) for Pb/PbO_2_, Ti/Sb-doped SnO_2_ and Si/BDD anodes is shown in Fig. 6. These last units are the most commonly used to comparison since the rate constants are normalized by the exposed area of the anodes and the current density applied. As expected a similar trend to that for RNO bleaching reaction was observed for the discoloration of MR solutions.

The corresponding oxidation curves clearly indicated that higher MR removal rate is achieved for Si/BDD and PbO_2_ anodes but not so for Ti/Sb-doped SnO_2_ electrode which is confirmed by comparing the corresponding values of the apparent rate constants derived from the kinetic analysis of the oxidation curves: 38.12, 11.88 and 0.53 L A^−1^ h^−1^ for Si/BDD, PbO_2_ and SnO_2_ anodes, respectively. Although these results clearly showed the effectivity trend of the anodes toward the MR oxidation, a better comparison parameter should be used for the values of electrocatalytic activity since it is one of the most important catalytic properties for catalytically active materials. Then, the electrocatalytic activity of the PbO_2_, Sb-doped SnO_2_ and BDD anodes for the oxidation of MR in 0.5 M H_2_SO_4_ was estimated as,^36^10
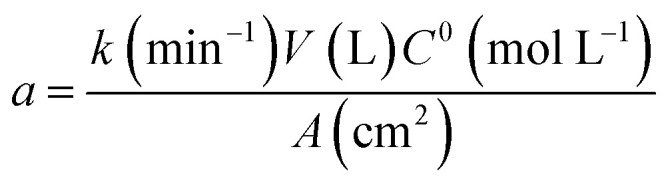
where, *k* is the apparent rate constant, *V* the volume of MR solution, *C*^0^ the initial MR concentration and *A* the surface area of the electrocatalytically active anode. The calculated *a* values from the corresponding *k* values in units of min^−1^ are: 13.8 × 10^−8^, 4.4 × 10^−8^ and 2 × 10^−8^ mol min^−1^ cm^−2^ for BDD, PbO_2_ and SnO_2_ anodes, respectively.

Considering the oxidation reaction of MR as

11 C_15_H_15_N_3_O_2(ac)_ + 38˙OH → 15CO_2(g)_ + H_2_O_(l)_ + 3HNO_3(ac)_ + 48H_(ac)_^+^ + 48e_(aux)_^−^

the theoretical specific electrical charge ([A h L^−1^]_theo_) required for the complete oxidation of 20 mg L^−1^ MR, can be estimated as follow:12
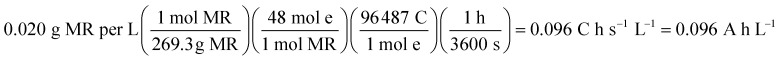


**Fig. 7:**
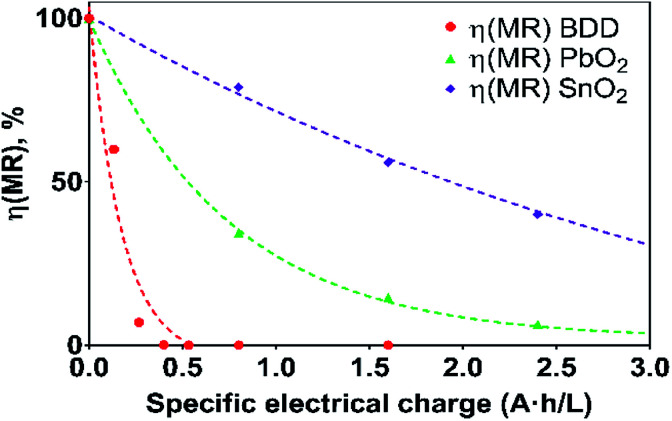
Current efficiency (*η*) values for the electrooxidation of MR as a function of the specific electrical charge (A h L^−1^) for solutions containing 20 mg L^−1^ MR in 0.5 M H_2_SO_4_ at 30 mA cm^−2^ and 298 K: 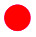 Si/BDD, 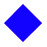 Ti/Sb-doped SnO_2_, 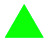 Pb/PbO_2_.

At 30 mA cm^−2^ and 120 min of electrolysis time, the experimentally required specific electrical charge ([A h L^−1^]_exp_) is about 2.4 A h L^−1^ for Ti/Sb-doped-SnO_2_ and 3.2 A h L^−1^ for Pb/PbO_2_, respectively; therefore, the current efficiency *η* (MR) for each value of the passed charge (*Q*) was calculated according to the following equation,^37^13
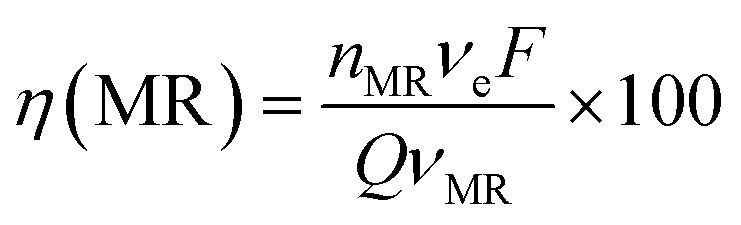
where *n*_MR_ is the number of moles of MR at *t* min of electrolysis time, *ν*_MR_ is 1, *ν*_e_ is 48 according chemical eqn (11), *F* is the Faraday constant (96 487 C mol^−1^) and *Q* is the circulated charge at the *t* electrolysis time in coulombs, Fig. 7.

As can be observed in Fig. 7, the current efficiency decreases with the passed charge because the MR concentration diminish and the intermediate organic compounds are oxidized a greater quantity; then, applied current is consumed by the parallel reaction, oxygen evolution (OER). Thus, under these conditions, the hydroxyl radicals produced on the anode by water discharge of chemical eqn (1) can react with MR causing its oxidation as shown the net chemical eqn (11), but also getting involved in the OER of eqn (4). These trends in current efficiency are expected since as overpotential for the OER increases with the type of anode used. The electrocatalytic activity to oxidized MR also enhances in the follow order: Si/BDD > Pb/PbO_2_ > Ti/Sb-doped SnO_2_, which confirms the high discoloration efficiency of the Si/BDD anodes and their quickly drop of current efficiency. As mentioned, the overpotential for OER and to the adsorption enthalpy of hydroxyl species on the anode surface, *i.e*., for a given anode material, the higher is the O_2_ overvoltage the higher is its oxidation power, are the main factors that determinates the electrooxidation capacity of non-active anodes. However, it was recently shown that BDD electrodes has also the capacity to produce S_2_O_8_^2−^ and SO_4_^−^˙ when sulfate is used as supporting electrolyte^38–40^ according to the reaction scheme (14):14
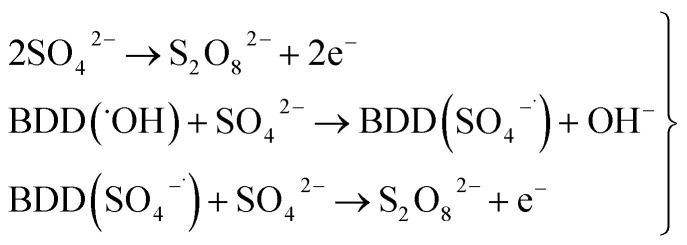


Therefore, the high participation of both ˙OH, S_2_O_8_^2−^ and SO_4_^−^˙ can to explain without doubt the fast discoloration of MR solutions and the best current efficiency observed for Si/BDD anode. Finally, this high capacity of Si/BDD anodes to discoloration of MR was supported by measuring the TOC at least three times of the discoloration reaction: TOC(MR)_*t*0_ = 12.5 ppm, TOC(MR)_*t*30_ = 6.1 ppm, TOC(MR)_*t*60_ = 4.0 ppm. These results clearly confirm that MR oxidation at Si/BDD anodes is efficiently achieved with high mineralization and color removals in short electrolysis time.

In the case of the Pb/PbO_2_ and Ti/Sb-doped SnO_2_ anodes, even when very similar overpotential for oxygen evolution (about 1.9 V/RHE *vs.* 2.1 V/RHE) were registered, Ti/Sb-doped SnO_2_ electrode exhibits slower MR discoloration rate and also lower current efficiency. This behavior is related to changes on the SnO_2_ surface structure under anodic polarization conditions; for instance, the hydration of the –Sn=O bond is achieved, and that the R pollutant is then adsorbed on the hydrated surface and, subsequently, oxidized by adsorbed ˙OH.^41^

### Electrocatalytic oxidation of 2,4-DNa

**Fig. 8:**
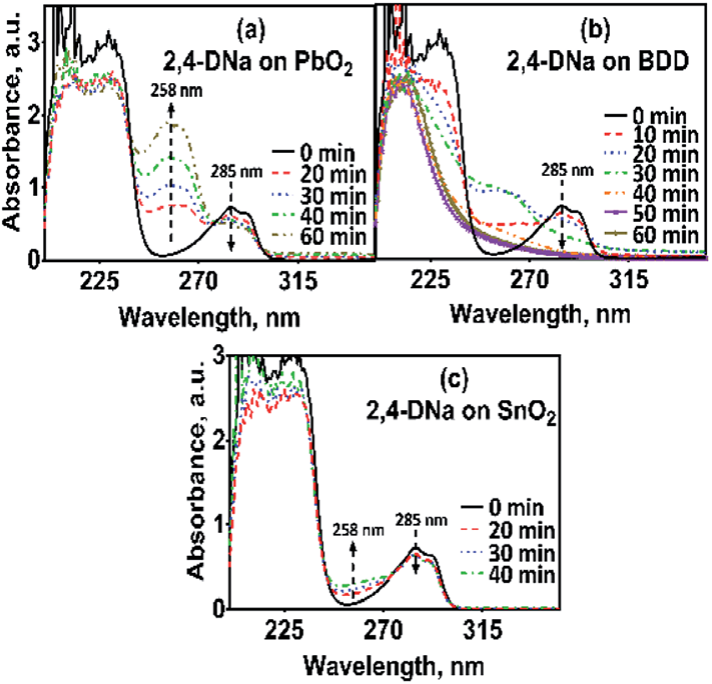
UV spectra for 100 mg L^−1^ 2,4-DNa solutions in 0.5 M Na_2_SO_4_ electrolyzed at 30 mA cm^−2^ and 298 K, as a function of the electrolysis time and the material of anode: (a) Pb/PbO_2_, (b) Si/BDD and (c) Ti/Sb-doped SnO_2_.

The electrooxidation of 2,4-DNa is certainly complicated in acidic medium and at the studied electrodes, as can be observed in Fig. 8. The spectroscopic UV profiles showed different band behaviors and oxidation levels of 2,4-DNa, depending on the anodes used. At Si/BDD electrodes (Fig. 8b), the wavelength range associated to the phenyl group (270–300 nm) disappears but not for the UV band at 210 nm (which can be related to the double bonds of the carboxylic acids formed during the electrocatalytic oxidation^42,43^). Additionally, a new UV band appeared at about 258 nm which reached a maximum absorbance at 30 min and then decreased, disappearing at 60 min of electrolysis time.15
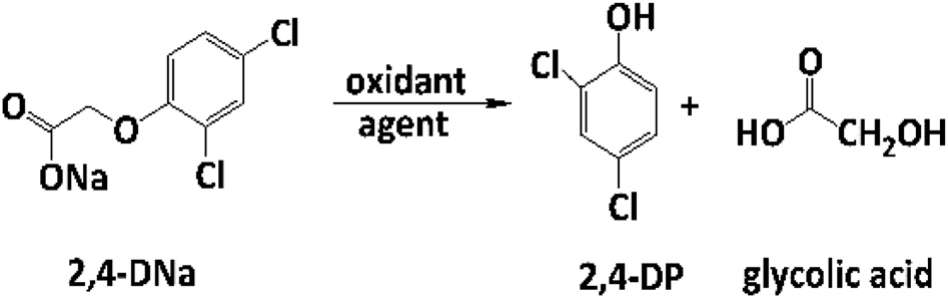


**Fig. 9:**
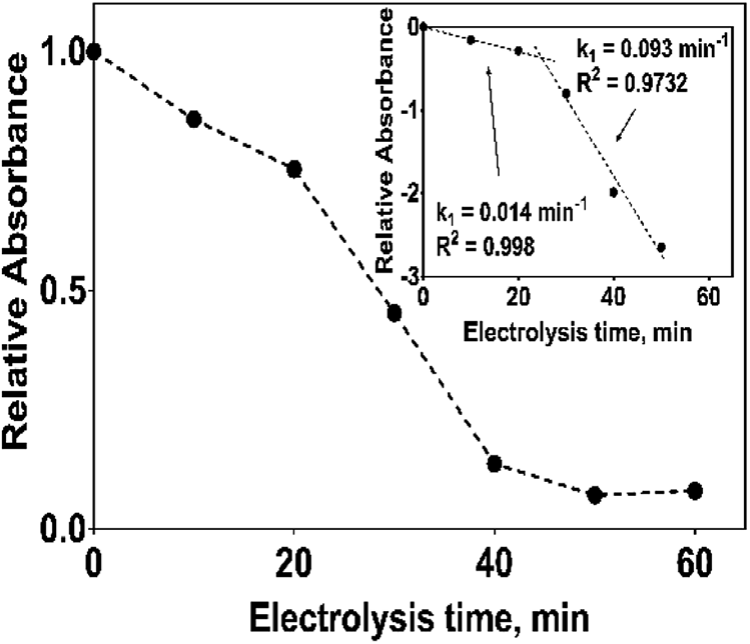
Plot of the relative absorbance at 285 nm of 2,4-DNa as a function of the electrolysis time for the electrolysis of a solution containing 100 mg L^−1^ 2,4-DNa in 0.5 M Na_2_SO_4_ at 30 mA cm^−2^ and 298 K on Si/BDD anode. Inset shows the corresponding pseudo-first order graphic.

The electrochemical oxidation of 2,4-DNa at Si/BDD electrodes showed an atypical behavior when the variation of the relative absorbance related to the phenyl group at 285 nm as a function of the electrolysis time, Fig. 9, is considered.

As can be observed, the relative absorbance decays in two segments of time with an inflection point at 30 min of electrolysis time. Each segment decreases with a kinetic of pseudo-first order (inset in Fig. 9): 0–25 min, *k* = 0.014 min^−1^ (*R*^2^ = 0.998); 25–50 min, *k* = 0.093 min^−1^ (*R*^2^ = 0.973). This kinetic behavior suggests that in the first 25 min of electrolysis time, the 2,4-DNa molecule breaks into two fragments as early oxidation product, 2,4-dichlorophenol (2,4-DP) and glycolic acid (2-hydroxyacetic acid), eqn (15). This assumption can be linked to the UV band at 258 nm which can be associated with the wavelength for the phenoxy-group (235 nm) of the 2,4-dichlorophenol (shifted in about 20 nm due the two chlorine atoms and the aqueous medium) formed during the first minutes of 2,4-DNa electrolysis as verified by the UV spectrum of pure compound and other published works.^26,44–46^ In order to support this assumption, the presence in solution at least of the 2,4-DP molecule was also confirmed by HPLC analysis in the first 15 min of electrolysis time (data not shown).

According to the obtained *k* values for 2,4-DNa oxidation, this first degradation stage is a presumably slow reaction due to the attack located on the weaker carbon of the aromatic ring (C1), since this C1 atom should be the site most energetically adequate to cleavage the 2,4-DNa molecule. Once the –C–O is broken and the 2,4-DP formed, the next oxidation stages take place quickly and the 2,4-DP molecule is degraded up to carboxylic acids and subsequently to CO_2_ and H_2_O.^47^ This result is in agreement with TOC measurements: TOC(2,4-DNa)_*t*0_ = 34 ppm, TOC(2,4-DNa)_*t*30_ = 2 ppm, TOC(2,4-DNa)_*t*60_ = 1 ppm.

**Fig. 10:**
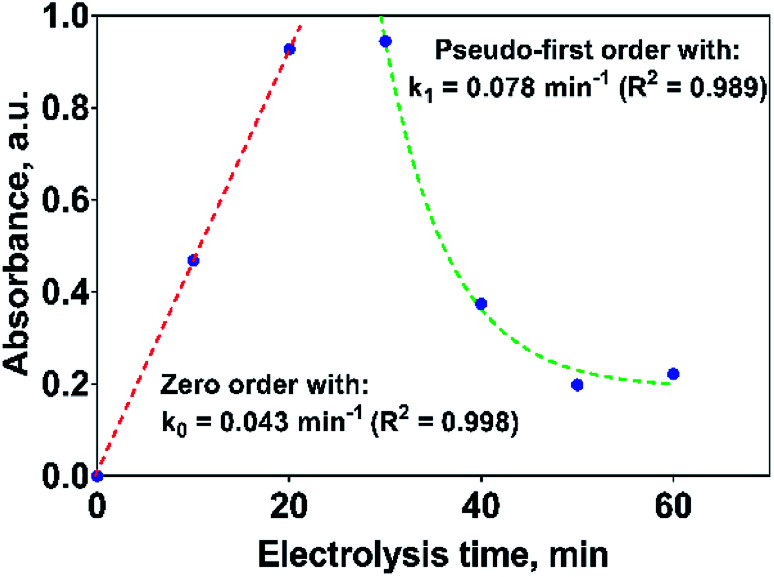
Kinetic parameters for the formation and consumption of 2,4-DP as first reaction intermediate on the Si/BDD anode in 0.5 M Na_2_SO_4_ + 100 ppm 2,4-DNa solution at 30 mA cm^−2^ and 298 K.

If the kinetic behavior of the formation and elimination of 2,4-DP is analyzed by using the absorbance data at 258 nm, Fig. 10; then, 2,4-DP is formed following a zero order reaction with a rate constant of *k*_0_ = 0.043 AU per min (*R*^2^ = 0.998), which is independent of the 2,4-DP concentration. Meanwhile, it is consumed through a pseudo-first order reaction with *k*_1_ = 0.078 min^−1^ (*R*^2^ = 0.989) as rate constant.

**Fig. 11:**
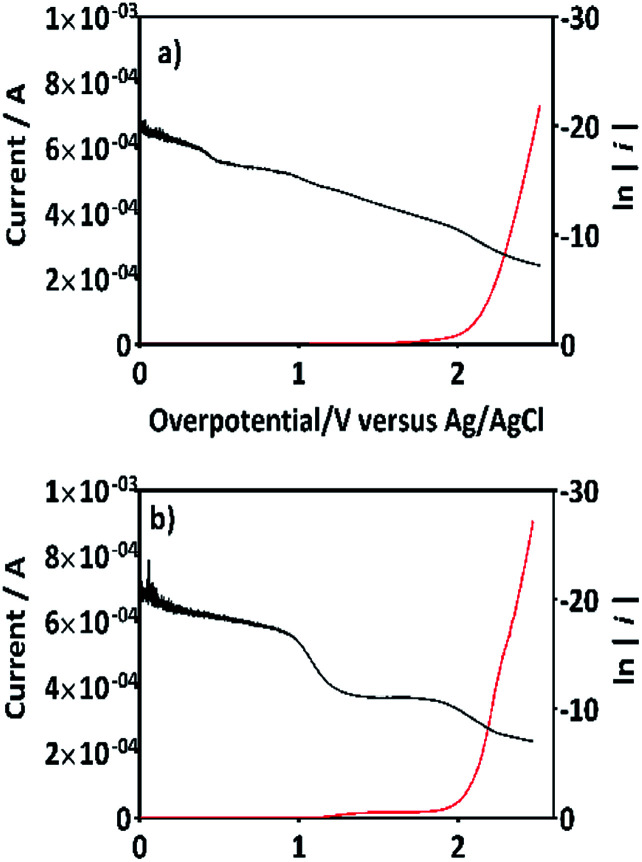
The variation of current as a function of the overpotential (*η*) for BDD in (a) 0.5 M Na_2_SO_4_ solution and (b) 0.5 M Na_2_SO_4_ + 100 ppm 2,4-DNa (pH 3) solution at 10 mV s^−1^ and 298 K. Tafel curves are included for each one of the cases plotting ln(*i*) against *η* (black curves).

If this proposal is viable, then, the formation of 2,4-DP (as intermediate) should be the result of an initial oxidation of 2,4-DNa directly on BDD surface which is induced by the action of SO_4_^−^˙ followed by a mediated oxidation by the ˙OH. This assumption is supported by the BDD polarization figures (including its Tafel curve) obtained in 0.5 M Na_2_SO_4_ and 0.5 M Na_2_SO_4_ + 100 ppm 2,4-DNa, Fig. 11.

The polarization curves showed two overpotential regions where chemical and/or electrochemical processes takes place involving electron transfers in some particular sequence, being more evident when 2,4-DNa was present in the electrolytic solution. In order to discuss each showed case, the Tafel parameters are summarized in Table 2, taking into account that these parameters were obtained from the Tafel equation expressed as,

16 *η* = *a* + *b* ln ∣*i*∣

where17
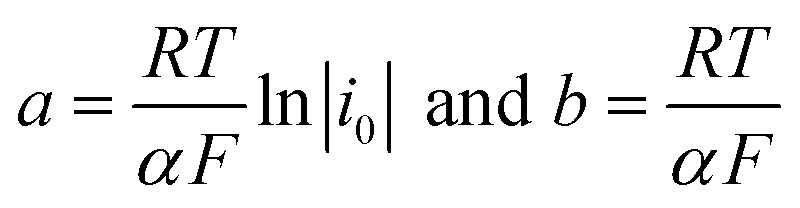


**Table 2:** Tafel parameters for Si/BDD anode estimated from the values of *a* and *b* of eqn (17)*via* Tafel plots of Fig. 11 and treated by eqn (16)

Parameters	0.5 M Na_2_SO_4_		0.5 M Na_2_SO_4_ + 100 ppm 2,4-DNa	
	*η* ≦ 1.5 V	*η* ≧ 2.0 V	*η* ≦ 1.5 V	*η* ≧ 2.0 V
*b* (mV)	219	125	53	113
*α*	0.117	0.205	0.49	0.226
*i*_0_ (A)	2.7 × 10^−9^	3.3 × 10^−12^	1.2 × 10^−18^	6.2 × 10^−14^

By using *R* = 8.314 J K^−1^ mol^−1^, *T* = 298 K and *F* = 96 487 J V^−1^ mol^−1^, then the Tafel slope reduces to *b* = (0.0257 V)*α*^−1^, from which both *α* and *i*_0_ can be calculated.

In the overpotential region between 2.0–2.5 V the polarization curve for Si/BDD in 0.5 M Na_2_SO_4_ showed a typical Tafel behavior associated to two electron transfer with a Tafel slope slightly shifted to 125 mV, a transfer coefficient of *α* = 0.205 and an exchange current of *i*_0_ = 3.3 × 10^−12^ A. The *α* and *i*_0_ values are in concordance with those reported for the oxygen evolution reaction (OER) at BDD in 0.25 M H_2_SO_4_ (*α* = 0.25; *i*_0_ = 5 × 10^−11^ A cm^−2^).^48^ According to the Comninellis' model,^15^ the OER at BDD can be written as (18):

18 2BDD(˙OH)_ads_ → O_2(g)_↑ + 2H_(ac)_^+^ + 2e_(aux)_ + 2BDD

Then, for BDD anode in 0.5 M Na_2_SO_4_ + 100 ppm 2,4-DNa solution (Fig. 11b), the polarization curve suggests the occurrence of an electron transfer process in addition to the OER and it could be associated to the oxidation of the organic compound. According to the *b* (113 mV) and *i*_0_ (6.2 × 10^−14^ A) values, it is possible to propose a reaction sequence of ÊE type (E, indicates an electrochemical step, and ^ indicates a rate-determining step) (19) being the Ê step the rate-determining step (rds),19
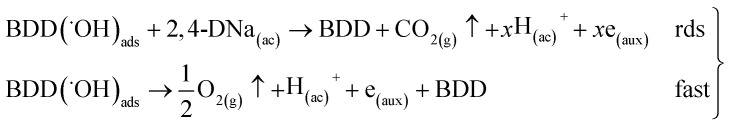


Hence, at over-potentials below 1.5 V, there are important differences between the two systems. For BDD in 0.5 M Na_2_SO_4_, the Tafel results suggest a water discharge step with formation of ˙OH radicals which are physically adsorbed on the BDD surface. Several cases involving hydrogen and/or oxygen adsorbed species have shown Tafel slopes *b* > 120 mV that depend on the coverage.^49^ In this way, the step (1) now raised how (20) using BDD.

20 BDD + H_2_O_(l)_ → BDD(˙OH)_ads_ + H_(ac)_^+^ + e_(aux)_^−^

is the associated step to the Tafel parameters of Table 2 for the 0.5 M Na_2_SO_4_ electrolytic medium. When 2,4-DNa is present in the electrolytic solution, Tafel parameters change significantly, the Tafel slope is ∼60 mV (real 53 mV) which indicate that two electrons reaction is involved in an EĈE (C indicates a chemical step) sequence (21), as those previously described for the MR discoloration,21
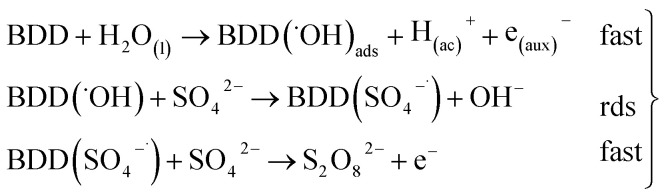


**Fig. 12:**
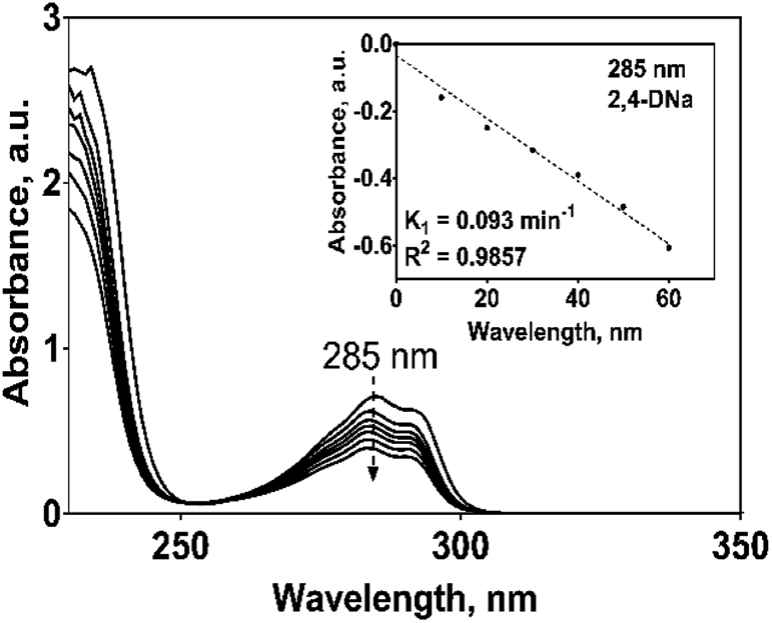
UV spectra, as a function of the electrolysis time, for 100 mg L^−1^ 2,4-DNa solutions in 0.5 M NaClO_4_ electrolyzed on a Si/BDD anode at 30 mA cm^−2^ and 298 K. Inset shows the pseudo-first order graphic of the UV band decrease at 285 nm.

The SO_4_^−^˙ formed in this overpotential region should be the oxidant agent mainly involved in the first fragmentation step of 2,4-DNa to form 2,4-DP. It could be considered as a direct oxidation on the BDD surface followed by a mediated oxidation by ˙OH radicals until the mineralization of 2,4-DP. An important feature to be highlighted about this model, is the fact that the SO_4_^−^˙ are formed on BDD anodes only in a sulfate medium (H_2_SO_4_ or Na_2_SO_4_).^40,50^ It was verified in 0.5 M NaClO_4_ solution (Fig. 12) where both the whole diminish of the UV spectra and the absence of UV bands around 250 nm are related to a combustion process of the organic compound on BDD anodes,^51^ in absence of sulfate-oxidizing species.

**Fig. 13:**
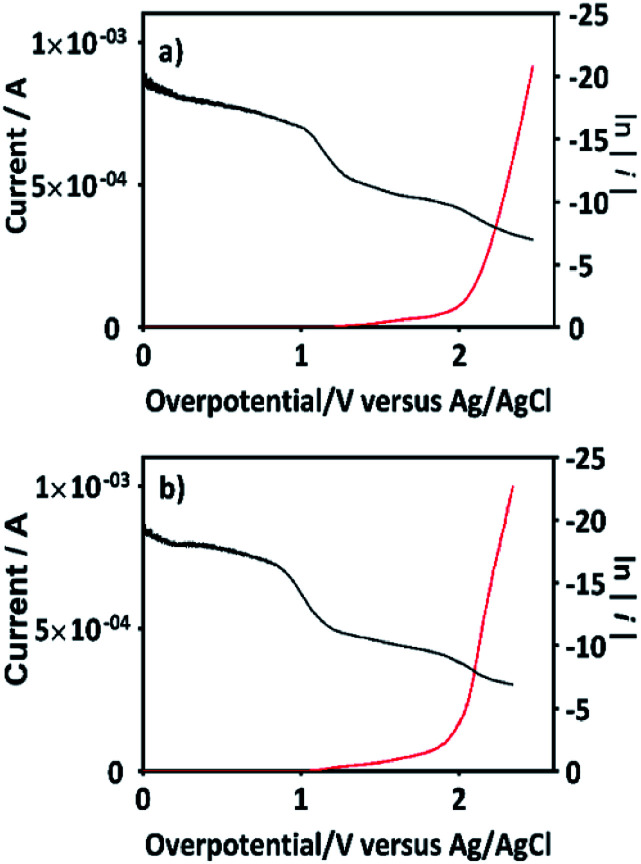
The variation of current as a function of the overpotential (*η*) for BDD in (a) 0.5 M NaClO_4_ solution and (b) 0.5 M NaClO_4_ + 100 ppm 2,4-DNa (pH 3) solution at 10 mV s^−1^ and 298 K. The Tafel analysis is included for each one case in the corresponding figure (plotting ln(*i*) against *η*).

An important aspect to be highlighted is that the apparent rate constant of pseudo-first order for the oxidation of 2,4-DNa at BDD in 0.5 M NaClO_4_ (*k*_1_ = 0.093 min^−1^) exactly matches with the apparent rate constant of pseudo-first order of the second segment of the oxidation curve of 2,4-DNa at BDD in sulfate medium (*k*_1_ = 0.093 min^−1^). This result validates the oxidation model presented for 2,4-DNa on BDD anode. In order to strengthen this proposal, the polarization curves of the BDD anode in 0.5 M NaClO_4_ with and without 2,4-DNa in solution have been obtained (Fig. 13a and b).

Additionally, Tafel parameters reported in Table 3 are consistent with the assertion on the mediated oxidation of 2,4-DNa by ˙OH until to its subsequent mineralization.

**Table 3:** Tafel parameters for BDD anode calculated from the values of *a* and *b* of eqn (18)*via* Tafel plots of Fig. 11 and treated by eqn (17)

Parameters	0.5 M NaClO_4_		0.5 M NaClO_4_ + 100 ppm 2,4-DNa	
	*η* ≦ 1.5 V	*η* ≧ 2.0 V	*η* ≦ 1.5 V	*η* ≧ 2.0 V
*b* (mV)	63	152	60	144
*α*	0.40	0.17	0.43	0.19
*i*_0_	1.2 × 10^−14^	1.5 × 10^−10^	4.3 × 10^−14^	1.6 × 10^−10^

**Fig. 14:**
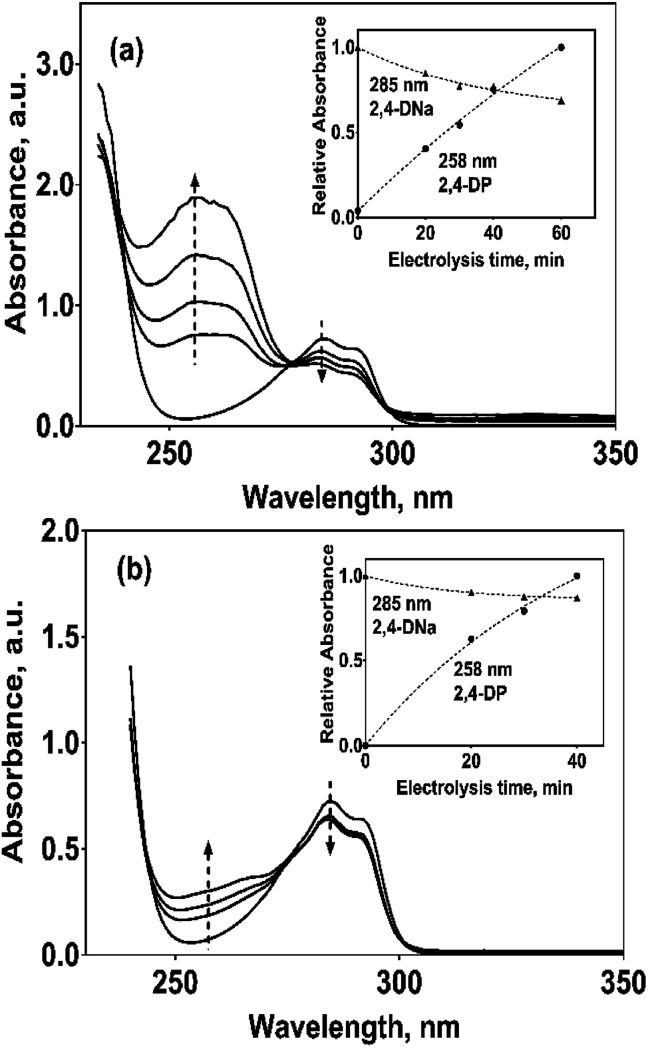
UV spectra for 100 mg L^−1^ 2,4-DNa solutions in 0.5 M Na_2_SO_4_ electrolyzed at 30 mA cm^−2^ and 298 K, as a function of the electrolysis time and on (a) Pb/PbO_2_ and (b) Ti/Sb-doped SnO_2_ anodes, respectively. The corresponding insets show the variation of the relative absorbance for both 258 and 285 nm UV bands.

In the case of the PbO_2_ and Sb-doped SnO_2_ anodes, UV spectra showed that a partial oxidation of 2,4-DNa was attained, producing 2,4-DP (Fig. 8a). A limited oxidation was achieved (45% and 13%) (Fig. 8c) using PbO_2_ and Sb-doped SnO_2_ anodes, respectively. At 60 min of electrolysis, only the formation of 2,4-DP was observed and it followed a zero order reaction with a *k*_0_ = 0.0302 AU per min (*R*^2^ = 0.993) for PbO_2_ and a *k*_0_ = 0.0056 AU per min (*R*^2^ = 0.999) for SnO_2_. However, no disappearance was noticed at least in the electrolysis time interval showed in Fig. 14a and b, respectively. This fact is not strange since in many works has been observed that the oxidation reaction usually takes place at longer times,^45–47,52^ depending on the method used to degrade 2,4-D (as acid) herbicide.

These results showed clearly that the oxidation of 2,4-DNa occurred with the participation of ˙OH, SO_4_^−^˙ and S_2_O_8_^2−^, considering that its conversion to 2,4-DP is the first oxidation step *via* SO_4_^−^˙ and S_2_O_8_^2−^ action. As previously reported,^53^ the participation of SO_4_^−^˙ and S_2_O_8_^2−^ is not restrictive to BDD anodes during the electrochemical oxidation of organic compounds and it can then be extended to Pb/PbO_2_ and Ti/Sb-doped SnO_2_ anodes. However, the S_2_O_8_^2−^ production could be only associated to the presence of hydroxyl radicals on the surface of anodes.^40,54^ Certainly, if the kinetic data for producing hydroxyl radicals on Pb/PbO_2_ and on Ti/Sb-doped SnO_2_ (Table 1) are compared, then, PbO_2_ is more efficient that SnO_2_, which also explain its electroactivity to favor the conversion from 2,4-DNa to 2,4-DP. This result is in agreement with the apparent rate constants above quoted.

## Conclusions

The outcomes presented here aim to demonstrate that the electrochemical oxidations of MR and 2,4-DNa are achieved using BDD, Pb/PbO_2_ and Ti/Sb-doped SnO_2_ (non-active anodes). It was showed that all anodes have a good capacity to produce hydroxyl radicals and therefore, all of them can efficiently degrade the organic pollutants, but in different oxidation levels. According to the *k*′ (in min^−1^) values, the electrocatalytic activity order should be Si/BDD > Pb/PbO_2_ > Ti/Sb-doped SnO_2_. Certainly, this electroactivity trend was clearly observed in the discoloration process of 0.5 M H_2_SO_4_ + 20 ppm MR solutions where a complete discoloration was achieved, in all cases. Meanwhile, a complete MR degradation was attained on Si/BDD anode in short electrolysis times, but longer treatment times are necessary to degrade MR efficiently on Pb/PbO_2_ and Ti/Sb-doped SnO_2_ anodes. The higher degradation capacity of Si/BDD anodes has been explained by a combined action of both ˙OH and SO_4_^−^˙ and S_2_O_8_^2−^ species. The efficacy of the *in situ* electrogeneration of SO_4_^−^˙ and S_2_O_8_^2−^ using BDD anodes has been well-established^13,55^ which depends on the ˙OH production and it could be a limiting factor on metallic oxides (MO_*x*_).^39^

The oxidation of 2,4-DNa showed to be a more complex process owing to the presence of the chlorine groups on the aromatic ring. The formation of 2,4-DP take place by means of a first attack of the SO_4_^−^˙ on the C1 atom of the 2,4-DNa molecule to form 2,4-DP and glycolic acid. After that, an oxidation with ˙OH is achieved to produce the corresponding carboxylic acids, in the case of BDD anode. The results of oxidation in 0.5 M NaClO_4_ as well as the Tafel analysis in both type of supporting electrolytes (0.5 M Na_2_SO_4_ and 0.5 M NaClO_4_) confirmed that statement. For Pb/PbO_2_ and Ti/Sb-doped SnO_2_ anodes, only the formation of 2,4-DP was observed in the period of electrolysis time established. However, the participation of the SO_4_^−^˙ and S_2_O_8_^2−^ in the formation of 2,4-DP cannot be underestimated because it was demonstrated that can be also formed on PbO_2_ surface.^53^ An interesting conclusion is that the kinetic analysis of the production of ˙OH radicals, formation of 2,4-DP and oxidation of 2,4-DNa followed the same trend in electrocatalytic activity, allowing to indicate that, specific mechanisms and interaction on anode surface can be attained depending on the nature of the anode material. Finally, the experimental results concerning the H_2_ production and the theoretical values which were estimated by the Faraday's law, respectively; fitted very well, showing that the hydrogen production rate *r*(H_2_) is independent of the nature of the anodic material even when an important effect on the O_2_ production was attained. However, these results are detailed discussed in the second part of this paper.

## Conflicts of interest

There are no conflicts to declare.

## Supplementary Material
